# Comparing cup placement, leg length, and offset discrepancy after total hip arthroplasty between CT-based robotic arm-assisted and navigation systems

**DOI:** 10.1302/2633-1462.61.BJO-2024-0173.R1

**Published:** 2025-01-01

**Authors:** Akira Shimizu, Satoshi Murakami, Takayuki Tamai, Yuuki Haga, Tatsuhiko Kutsuna, Tomofumi Kinoshita, Masaki Takao

**Affiliations:** 1 Department of Orthopedic Surgery, Ozu Memorial Hospital, Ozu, Japan; 2 Department of Orthopaedic Surgery, Ehime University Graduate School of Medicine, Toon, Japan

**Keywords:** Total hip arthroplasty, Robotic arm-assisted system, CT-based navigation system, Leg length discrepancy, Offset discrepancy, total hip arthroplasty (THA), robotic arms, leg length, hips, propensity score matching, hip osteoarthritis, Orthopedic Surgery, acetabular component position, hip joints, developmental dysplasia of the hip (DDH)

## Abstract

**Aims:**

Excellent outcomes have been reported following CT-based robotic arm-assisted total hip arthroplasty (rTHA) compared with manual THA; however, its superiority over CT-based navigation THA (nTHA) remains unclear. This study aimed to determine whether a CT-based robotic arm-assisted system helps surgeons perform accurate cup placement, minimizes leg length, and offsets discrepancies more than a CT-based navigation system.

**Methods:**

We studied 60 hips from 54 patients who underwent rTHA between April 2021 and August 2023, and 45 hips from 44 patients who underwent nTHA between January 2020 and March 2021 with the same target cup orientation at the Department of Orthopedic Surgery at Ozu Memorial Hospital, Japan. After propensity score matching, each group had 37 hips. Postoperative acetabular component position and orientation were measured using the planning module of the CT-based navigation system. Postoperative leg length and offset discrepancies were evaluated using postoperative CT in patients who have unilateral hip osteoarthritis.

**Results:**

The absolute differences in radiological inclination (RI) and radiological anteversion (RA) from the target were significantly smaller in rTHA (RI 1.2° (SD 1.2°), RA 1.4° (SD 1.2°)) than in nTHA (RI 2.7° (SD 1.9°), RA 3.0° (SD 2.6°)) (p = 0.005 for RI, p = 0.002 for RA). The absolute distance of the target’s postoperative centre of rotation was significantly smaller in the mediolateral (ML) and superoinferior (SI) directions in rTHA (ML 1.1 mm (SD 0.8), SI 1.3 mm (SD 0.5)) than in nTHA (ML 1.9 mm (SD 0.9), SI 1.6 mm (SD 0.9)) (p = 0.002 for ML, p = 0.042 for SI). Absolute leg length and absolute discrepancies in the acetabular, femoral, and global offsets were significantly lower in the rTHA group than in the nTHA group (p = 0.042, p = 0.004, p = 0.003, and p = 0.010, respectively). In addition, the percentage of hips significantly differed with an absolute global offset discrepancy of ≤ 5 mm (p < 0.001).

**Conclusion:**

rTHA is more accurate in cup orientation and position than nTHA, effectively reducing postoperative leg length and offset discrepancy.

Cite this article: *Bone Jt Open* 2024;6(1):3–11.

## Introduction

Outcomes following total hip arthroplasty (THA) in patients who have osteoarthritis (OA) are affected by the cup position and orientation. An improper cup position can lead to aseptic loosening, psoas tendinitis, leg length discrepancy (LLD), and unintended bone loss. In addition, improper cup alignment can lead to mechanical complications associated with impingement and/or edge-loading, including dislocation, excessive wear, and breakage of bearing materials and components.^1-3^ Postoperative limb offset discrepancies significantly affect the soft-tissue tension in THA.^4^ Similarly, marked LLD after THA contributes to gait asymmetry, knee and back pain, abnormal force transmission across the hip, and revision surgery.^5,6^

Computer-assisted surgical (CAS) systems, including navigation and robotics, have reportedly reduced cup malorientation and early mechanical failure during THA.^7,8^ The benefits of using the CAS system for leg length and offset management have also been shown.^9-12^ Notably, CT-based systems, including robotic arms and navigation systems, have been shown to improve cup position and orientation accuracy.^13-15^ Consequently, several studies have compared the accuracy of cup position and orientation between CT-based robotic arm-assisted THA (rTHA) and CT-based navigation THA (nTHA), and rTHA showed superior accuracy in cup orientation.^16-19^ However, the superiority of cup position accuracy in rTHA is controversial, regardless of the strict robot-arm control of the reamed bony area. It is also unclear whether there are any differences in the postoperative leg length and offset discrepancies between rTHA and nTHA.

Therefore, this study aimed to determine whether a CT-based robotic arm-assisted system allows surgeons to perform accurate cup placement, minimize leg length, and offset discrepancies more than a CT-based navigation system.

## Methods

### Patients

The ethics committee of Ozu Memorial Hospital, Japan, approved this retrospective case-control study. THA was performed using a CT-based hip navigation system (Stryker CT-Hip System v. 1.1; Stryker, Germany) in 45 joints from 44 patients between January 2020 and March 2021 and 60 joints from 54 patients using a CT-based robotic arm-assisted system (Mako Total Hip; Stryker, USA) between April 2021 and August 2023. Only a single CT-based scan was performed during each period. Notably, all patients were preoperatively diagnosed with primary OA or secondary OA to developmental dysplasia of the hip (DDH). OA secondary to DDH was classified into group I or II based on Crowe et al’s^20^ classification system. Propensity score matching was used to match the patients by age, sex, and BMI between the two groups using EZR (v. 1.61 (Jichi Medical University, Japan)).^21^ Overall, 37 hips from each group were included. The groups did not significantly differ in height, weight, treated side, diagnosis of hip disorders (primary OA or OA secondary to DDH), or surgical approach. Table I shows the patient demographic details.

**Table I.** Patient demographic details.

Variable	rTHA (37 hips)	nTHA (37 hips)	p-value
Mean age, ys (SD)	68.8 (10.6)	69.5 (9.0)	0.759*
Sex (female/male), n (%)	25/12 (68/32)	26/11 (70/30)	0.802†
Mean height, cm (SD)	153.7 (9.2)	154.7 (11.3)	0.688*
Mean weight, kg (SD)	60.3 (10.0)	59.4 (12.5)	0.756*
Mean BMI, kg/m^2^ (SD)	25.5 (3.6)	24.7 (4.2)	0.391*
Treated side (right/left), n (%)	18/19 (49/51)	19/18 (51/49)	0.816†
**Diagnosis**			0.809‡
Primary OA, n (%)	13 (35)	14 (38)	
DDH (Crowe classification I/II), n (%)	24 (22/2) (65)	23 (19/4) (62)	

* Mann-Whitney U test.

† Chi-squared test.

‡ Chi-squared test compared with primary OA and DDH.

DDH, developmental dysplasia of the hip; nTHA, CT-based navigation total hip arthroplasty; OA, osteoarthritis; rTHA, CT-based robotic arm-assisted total hip arthroplasty.

### Preoperative cup planning

Preoperative CT images of the bilateral hip joints were obtained from the iliac wing to the femoral condyle using a 16-row multi-slice CT scanner (Aquilion 16; Canon, Japan) with a 1 mm slice pitch. Then, the CT data were transferred to the planning module of the CT-based hip navigation system (Stryker). For preoperative acetabular component planning, a functional pelvic coordinate system was used for the rTHA and nTHA groups following a method reported by Sugano.^22^ Notably, the retrocondylar coordinate system was used in both groups as the femoral coordinate system, as previously described.^4,22^

**Fig. 1:**
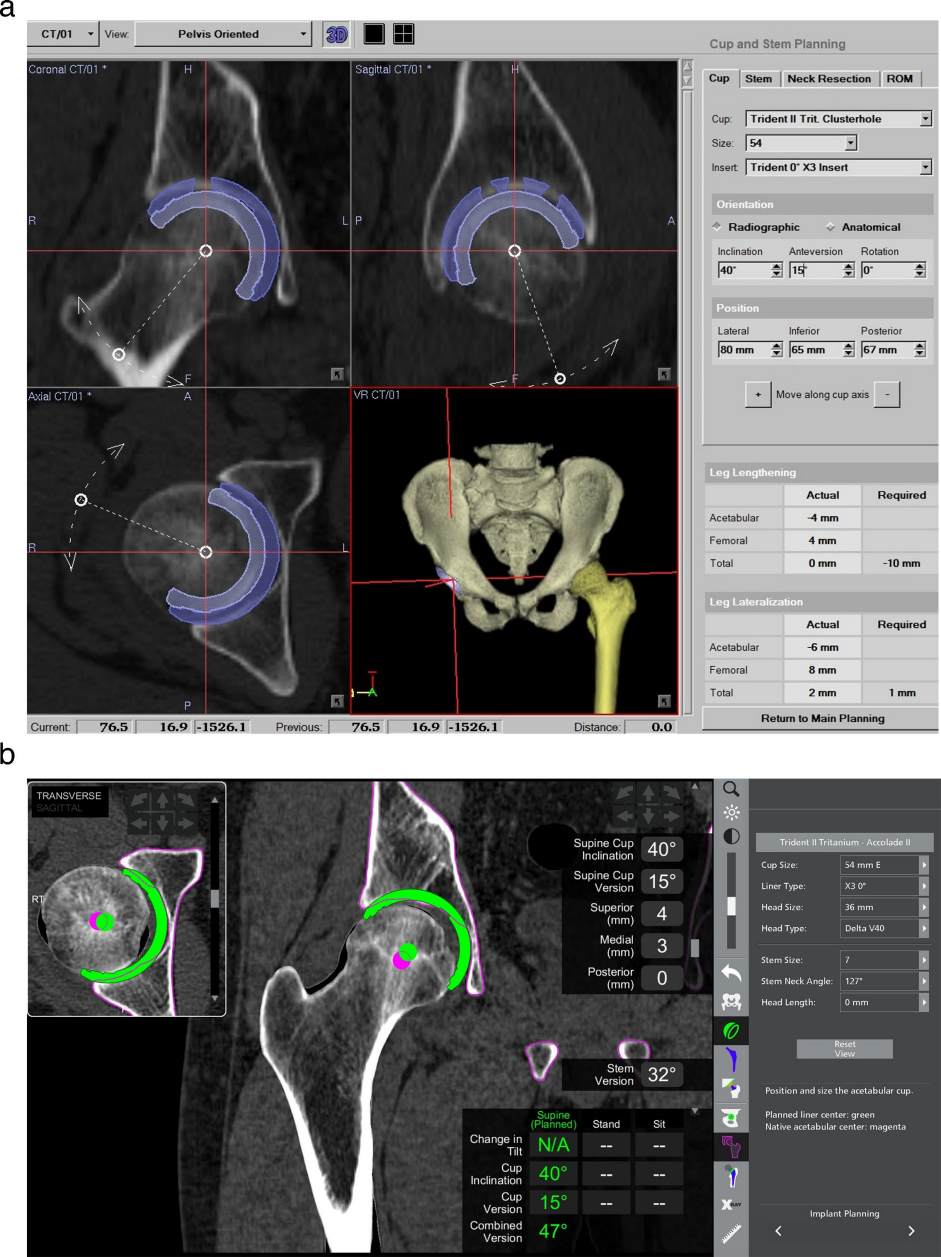
Preoperative planning was performed with a) a CT-based hip navigation system (Stryker CT-Hip System v. 1.1; Stryker, Germany), and then b) the data were accurately transferred to the Mako Total Hip application (Stryker, USA).

In both groups, we determined the target cup angle at 40° radiological inclination (RI) and 15° radiological anteversion (RA). In the rTHA group, the landmark positions for constructing pelvic and femoral coordinates, as well as the cup and stem placement positions, were transferred into the Mako Total Hip (Stryker) application by the technicians under visual comparison of the two planning monitors. The accuracy of plan transfer using this method has been reported to be 0.5° in cup alignment (Figure 1).^23^

### Surgical procedure

Three senior surgeons (AS, SM, MT) performed all procedures using two types of anterolateral approaches (the modified Watson-Jones and mini-incision anterolateral approaches) with the patients in the lateral position.^24,25^ A mini-incision anterolateral approach was employed for patients with severe obesity or significant deformities in the hip joints. Two cementless cups (Trident and Trident II; Stryker, USA) were used. Screw fixation was performed based on the surgeon’s judgement of the strength of press-fit cup fixation, and a cementless stem (Accolade II; Stryker) was manually implanted in all the cases.

### rTHA

Three pins were placed in the superolateral aspect of the iliac crest to secure the pelvic array. Before dislocation of the hip, the femoral marker screw was inserted in the great trochanter and the femoral position was recorded by registering the femoral marker screw and the lateral epicondyle of the knee with the hip joint in the neutral position and the knee joint in flexion at 90°. The position of the femoral neck resection was determined by measuring the distance between the tip of the femoral head and the planned line of the femoral neck resection. After acetabular registration, one-size under or same-size reaming was performed in all cases. The cup was placed in the reamed position at the planned angle under robotic arm guidance. A cementless stem was manually implanted. The intraoperative change and discrepancy in leg length and offset were measured by registering the femoral marker screw and the knee’s lateral epicondyle with the hip joint in the neutral position and the knee joint in 90° flexion (the so-called ‘express mode’), and the head offset was determined.

### nTHA

After placing the pelvic array on the superolateral aspect of the iliac crest, surface registration of the pelvis was performed using four landmarks and 30 points on the periacetabular area. Four landmarks were determined during preoperative planning: the anterior superior iliac spine, innominate tuberosity of the iliac crest, and the anterior and superior edges of the acetabulum. Three steps of acetabular reaming were performed in the planned position, and one under-reaming step was adopted. The acetabular component was implanted at the planned location and aligned under the navigation system’s real-time guidance. A cementless stem was implanted manually. The intraoperative leg length and offset were evaluated by referencing the preoperative plan, and the head offset was determined.

### Postoperative evaluation

Postoperative pelvic CT was acquired two to six weeks postoperatively, with patient consent. The postoperative cup position and orientation were measured using the planning module of a CT-based navigation system (Stryker). After all reference landmarks on the preoperative CT-based plan were manually copied onto the postoperative CT images, computer-aided design models of the acetabular cups were superimposed on their images, as previously reported.^26^ The measurement accuracy of cup orientation difference between preoperative CT-based plan and postoperative CT was reported to be 0.5° using this method.^23^

The postoperative cup orientation (RI and RA) was measured, and the difference in angle between the postoperative and target cup orientations was investigated. The absolute difference in angle between the postoperative and target cup orientations was also evaluated. The number of cases with cup orientation outliers greater than 5° from the target was investigated.

Each axis’s distance of the postoperative centre of rotation (COR) from the target COR (mediolateral (ML), anteroposterior (AP), and superoinferior (SI) axes for each CT slice) was measured by defining the lateral, posterior, and superior directions as positive. The absolute distance between postoperative and target COR levels was also investigated. The 3D distance from the target to the postoperative COR was also calculated. The cup centre-edge angle (cup-CE angle) was measured as previously described.^27^

**Fig. 2:**
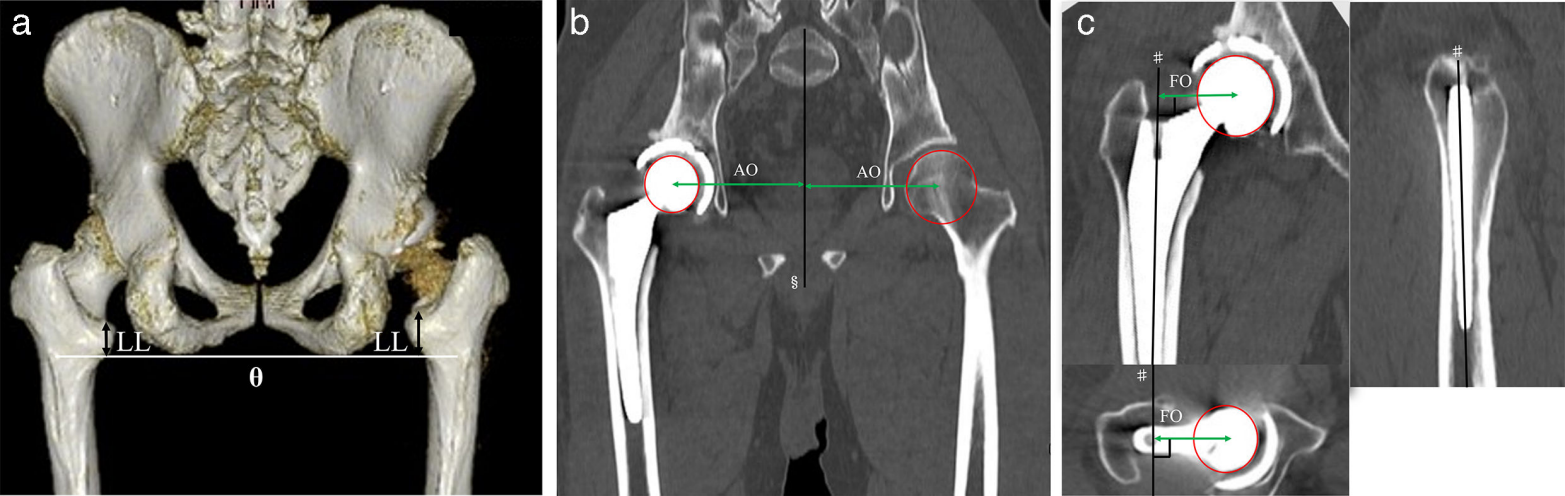
a) Pre- and postoperative leg length (LL), b) postoperative acetabular offset (AO) and femoral offset (FO), and c) global offset (AO + FO). Discrepancies were investigated using 3D image processing workstation (Ziostation 2; Ziosoft, Japan). θ: the line passing through bilateral distal ends of ischial tuberosities. §: the line passing through the centre of the first sacral vertebral body and the midpoint of bilateral pubic tubercles. ♯: the axis of the femoral canal.

Preoperative and postoperative LLD and postoperative offset discrepancy between the operative and contralateral sides were evaluated in patients who have unilateral hip OA, including 24 and 22 hips who underwent rTHA and nTHA, respectively. Leg length and offset were measured using a 3D image-processing workstation (Ziostation 2; Ziosoft, Japan) on preoperative and postoperative CT. The leg length was measured as the distance from the line passing through the bilateral distal ends of the ischial tuberosity to the upper margin of the lesser trochanter (Figure 2a). The LLD and absolute values were calculated. The percentage of cases with postoperative LLD within approximately 5 mm and approximately 10 mm was investigated. The acetabular offset was defined as the distance from the centre of the femoral or ceramic head to the line passing through the centre of the first sacral vertebral body and the midpoint of the bilateral pubic tubercles (Figure 2b). The femoral offset was defined as the perpendicular distance between the femoral canal’s axis, which was placed in the centre of the femoral metaphysis for the three planes, and the centre of the femoral or ceramic head (Figure 2c).^28^ Global offset was defined as the sum of the acetabular and femoral offsets. The lateral direction was defined as positive. Discrepancies in acetabular, femoral, and global offsets and their absolute values were investigated. The percentage of cases with postoperative global offset discrepancy within approximately 5 mm was also investigated.

The cup size, presence of screw fixation, and number of screws were evaluated. Operating time and total perioperative blood loss were also evaluated.

### Statistical analysis

Data in tables are expressed as means and SDs. Normal distribution was assessed using the Shapiro-Wilk test to compare continuous variables. Independent-samples *t*-test was performed for those with a normal distribution and the Mann-Whitney test for those without. The chi-squared test was used to compare the patients’ sex, treated side, and diagnosis. Other categorical data were analyzed using Fisher’s exact test, following Cochran’s rule.^29^ Statistical significance was set at a p-value < 0.05. All statistical analyses were performed using EZR software.

The sample size was calculated using the Power and Sample Size Calculation software v. 3.1.2 (Vanderbilt University, USA). As for cup position, the calculation was based on a previous study, in which cup positional accuracy within each group was normally distributed, with a standard deviation of 1.5 mm.^16^ If the true difference in cup positional accuracy between rTHA and nTHA was 1 mm, we would need to study 36 participants in each group to reject the null hypothesis that the population means of both groups are equal with a probability (power) of 0.8. The type I error probability associated with this null hypothesis test was 0.05. As for cup orientation, the calculation was based on the same study in which cup orientation accuracy within each group was normally distributed, with a standard deviation of 2°.^16^ If the true difference in cup orientation accuracy between rTHA and nTHA was 2.5°, we would need to study 28 participants in each group to reject the null hypothesis that the population means of both groups are equal with a probability (power) of 0.8. The type I error probability associated with this null hypothesis test was 0.05. As for LLD, the calculation was based on another study in which LLD within each group was normally distributed, with a standard deviation of 2.4 mm.^30^ If the true difference in cup positional accuracy between rTHA and nTHA was 2 mm, we would need to study 23 participants in each group to reject the null hypothesis that the population means of both groups are equal with a probability (power) of 0.8. The Type I error probability associated with this null hypothesis test was 0.05.

## Results

### Accuracy of cup orientation

**Fig. 3:**
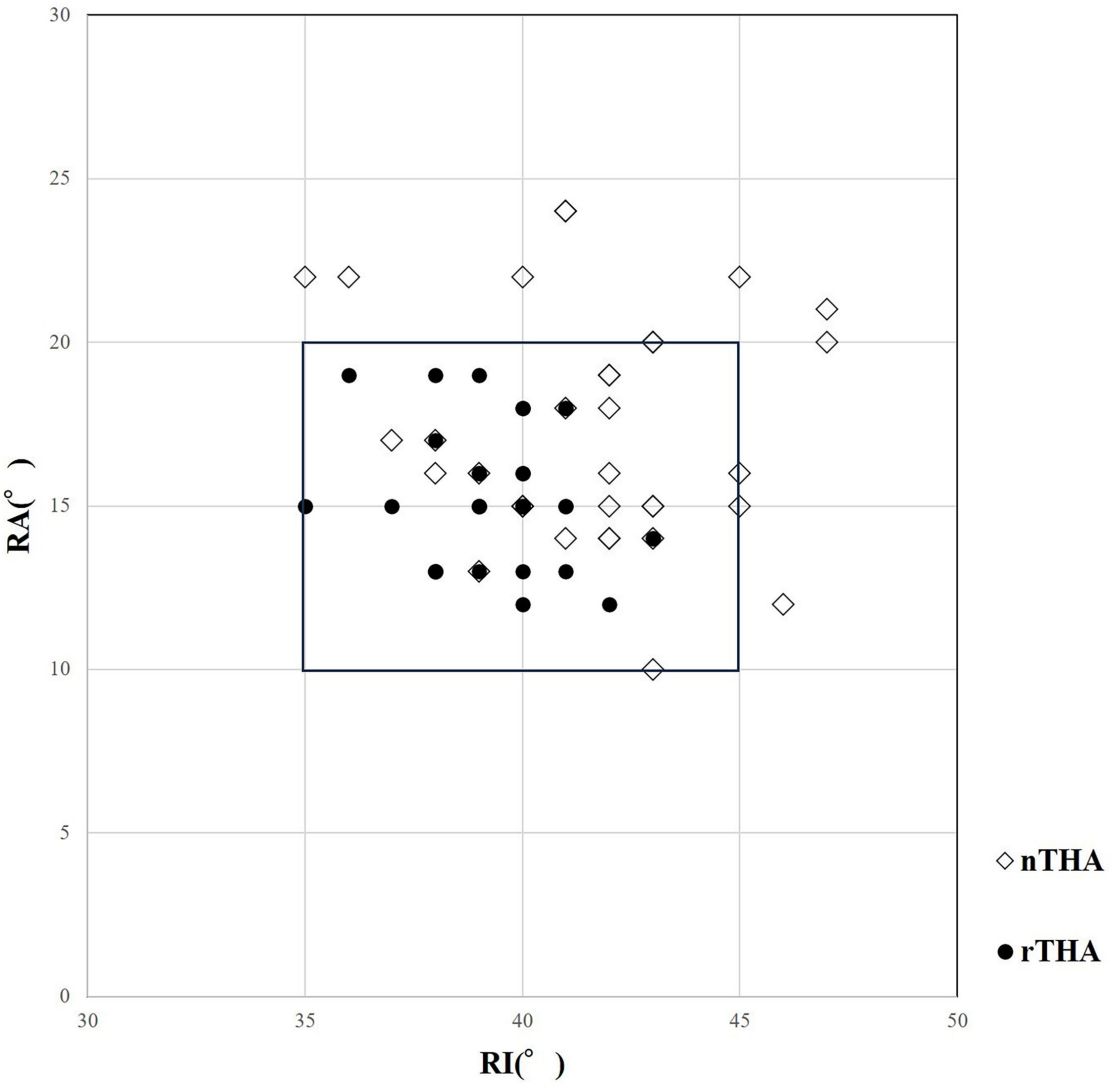
The result of postoperative radiological anteversion (RA) and radiological inclination (RI) was compared between CT-based robotic arm-assisted total hip arthroplasty (rTHA) and CT-based navigation total hip arthroplasty (nTHA) using scatterplots. The rectangle shows the ± 5° reconstruction zone.

The RI and RA were higher in the nTHA group (RI 41.4° (SD 2.9°), RA 17.1° (SD 3.4°)) than in the rTHA group (RI 39.6° (SD 1.7°), RA 15.3° (SD 2.1°)) (p = 0.002 for RI and p = 0.027 for RA). The absolute differences in the RI and RA from the target were significantly smaller in the rTHA group (ΔRI 1.2° (SD 1.2°), ΔRA 1.4° (SD 1.2°)) than in the nTHA group (ΔRI 2.7° (SD 1.9°), ΔRA 3.0° (SD 2.6°)) (p = 0.005 for ΔRI and p = 0.002 for ΔRA). The number of cases with cup orientation outliers greater than 5° from the target was significantly lower in rTHA (zero hips) compared to that in nTHA (eight hips) (p = 0.002) (Figure 3).

### Accuracy of cup position

The distance of the postoperative COR position from the target was not significantly different between the groups in the ML, AP, and SI directions. The positive or negative directions of the postoperative COR position from the target were similar between groups in the ML, AP, and SI directions. The absolute distance between the postoperative COR position and target was significantly smaller in the ML and SI directions than in the rTHA group (p = 0.002 for ΔML and p = 0.042 for ΔSI). The 3D distance between the postoperative COR position and the target was also significantly smaller in the rTHA group (p < 0.001) (Table II).

**Table II.** Comparison of the accuracy of acetabular component orientation and position between rTHA and nTHA.

Variable	rTHA (37 hips)	nTHA (37 hips)	p-value
**Mean postoperative cup orientation, ° (SD)**			
RI	39.6 (1.7)	41.4 (2.9)	0.002*
RA	15.3 (2.1)	17.1 (3.4)	0.027*
**Mean absolute difference between postoperative and target cup orientation, ° (SD)**			
ΔRI	1.2 (1.2)	2.7 (1.9)	0.005†
ΔRA	1.4 (1.2)	3.0 (2.6)	0.002†
**Mean distance of postoperative COR from the target, mm (SD)**			
ΔML	0.2 (1.2)	0.3 (2.0)	0.847*
ΔAP	-0.1 (1.5)	-0.7 (1.4)	0.134*
ΔSI	0.5 (1.4)	1.1 (1.5)	0.059*
**Mean absolute distance of postoperative COR from the target, mm (SD)**			
ΔML	1.1 (0.8)	1.9 (0.9)	0.002†
ΔAP	1.2 (0.8)	1.4 (0.8)	0.490†
ΔSI	1.3 (0.5)	1.6 (0.9)	0.042†
3D distance	2.2 (0.9)	3.0 (0.9)	< 0.001*

* Independent-samples *t*-test.

† Mann-Whitney U test.

AP, distance in anteroposterior direction; COR, centre of rotation; ML, mediolateral direction; nTHA, CT-based navigation total hip arthroplasty; RA, radiological anteversion; RI, radiological inclination; rTHA, CT-based robotic arm-assisted total hip arthroplasty; SI, distance in superoinferior direction.

### Leg length and offset discrepancy

The mean preoperative LLD was 7.8 mm (SD 6.9) and 7.6 mm (SD 6.0) in the rTHA and nTHA groups, respectively, with no significant difference between the groups (p = 0.878). There was no significant difference in postoperative LLD between the groups, whereas the postoperative absolute LLD was significantly lower in the rTHA group (p = 0.042). However, there was no significant difference in the percentage of hips with an absolute postoperative LLD ≤ 5 mm and 10 mm.

**Fig. 4:**
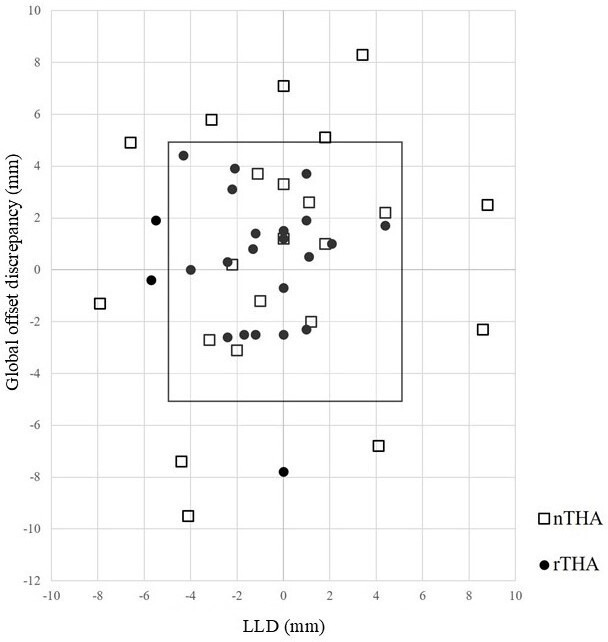
The result of postoperative leg length discrepancy (LLD) and global offset discrepancy was compared between CT-based robotic arm-assisted total hip arthroplasty (rTHA) and CT-based navigation total hip arthroplasty (nTHA) using scatterplots. The rectangle shows the ± 5 mm reconstruction zone.

The groups did not significantly differ in the postoperative acetabular, femoral, and global offset discrepancy. The absolute discrepancy of the postoperative acetabular, femoral, and global offsets was lower in the rTHA group (p = 0.004, p = 0.003, and p = 0.010 for the acetabular, femoral, and global offsets, respectively). In addition, there was a significant difference in the percentage of hips with a postoperative absolute global offset discrepancy of ≤ 5 mm (p < 0.001) (Table III). The number cases with both the postoperative LLD and global offset discrepancy within approximately 5 mm was 87% (21 of 24) for rTHA and 50% (11 of 22) for nTHA (p = 0.007) (Figure 4).

**Table III.** Comparison of leg length and offset discrepancies between rTHA and nTHA for patients who have unilateral hip osteoarthritis.

Variable	rTHA (24 hips)	nTHA (22 hips)	p-value
Mean preoperative LLD, mm (SD)	7.8 (6.9)	7.6 (6.0)	0.878*
Mean postoperative LLD, mm (SD)	-1.2 (3.1)	-0.1 (4.3)	0.261*
Mean postoperative absolute LLD, mm (SD)	1.9 (1.8)	3.3 (2.8)	0.042*
Hips with postoperative LLD of ≤ ± 5 mm, n (%)	22 (91.7)	18 (81.8)	0.405†
Hips with postoperative LLD of ≤ ± 10 mm, n (%)	24 (100)	22 (100)	1.000†
**Mean postoperative leg offset discrepancy, mm (SD)**			
Acetabular offset	-2.1 (1.9)	-3.1 (4.1)	0.297*
Femoral offset	2.4 (2.2)	3.8 (4.2)	0.182‡
Global offset	0.3 (2.7)	0.5 (4.7)	0.848‡
**Mean postoperative absolute leg offset discrepancy, mm (SD)**			
Acetabular offset	2.2 (1.8)	4.5 (3.1)	0.004*
Femoral offset	2.8 (0.5)	4.9 (2.7)	0.003‡
Global offset	2.1 (1.7)	3.8 (2.6)	0.010‡
Hips with global offset of ≤ ± 5 mm, n (%)	23 (95.8)	15 (68.2)	< 0.001†

* Mann-Whitney U test.

† Fisher’s exact test.

‡ Independent-samples *t*-test.

LLD, leg length discrepancy; nTHA, CT-based navigation total hip arthroplasty; rTHA, CT-based robotic arm-assisted total hip arthroplasty.

### Comparison of other surgical data

There were no significant differences between the groups in the surgical approach, surgical time, total perioperative blood loss, cup size, or cup-CE angle. The number of cases in which screw fixation was performed was significantly higher in the nTHA group (p < 0.001). The number of screws used per cup was also significantly higher in nTHA (p < 0.001) (Table IV).

**Table IV.** Comparison of surgical data between rTHA and nTHA.

Variable	rTHA (37 hips)	nTHA (37 hips)	p-value
**Surgical approach, n (%)**			1.000*
Modified Watson Jones	33 (89)	34 (92)	
Mini-incision anterolateral	4 (11)	3 (8)	
Mean surgical time, mins (SD)	91.5 (17.6)	88.1 (22.0)	0.471†
Mean total perioperative blood loss, g (SD)	520.0 (295.4)	482.4 (258.5)	0.562†
Mean cup size, mm (SD)	50.1 (3.0)	50.4 (2.9)	0.534†
Cups with screw fixation, n (%)	17 (46)	37 (100)	< 0.001*
Mean number of screws used per cup (SD)	1.6 (0.7)	2.2 (0.5)	< 0.001†
Mean cup CE angle,° (SD)	26.4 (7.2)	28.6 (8.1)	0.219†

* Fisher’s exact test.

† Mann-Whitney U test.

rTHA, robotic-assisted total hip arthroplasty; nTHA, navigation total hip arthroplasty; CE, centre-edge.

## Discussion

This study showed that rTHA was significantly more accurate than nTHA regarding absolute error to the target in cup orientation and positioning. Regarding cup positioning, superiority was observed in the ML and SI directions, resulting in lower absolute LLD and absolute leg offset discrepancy for rTHA in patients who have unilateral hip OA.

A valuable finding of this study was the cup positional error between the two groups. The absolute cup positional errors were smaller in the ML and SI directions, and there was no consistent trend in the positive or negative direction of error, indicating that robot arm-assisted reaming and cup implantation effectively reduced variation in the cup’s positioning error from the target position. Ando et al^16^ reported that cup positioning was more accurate in rTHA than in nTHA in the absolute AP and SI directions in the same CT-based evaluation, and that there was no consistent trend in the positive or negative error directions. Their results were consistent with our results regarding cup positioning accuracy in the SI direction. In rTHA, acetabular reaming is confined to the area created by the haptic boundary system and the robotic arm-controlled cup implantation. In nTHA, the surgeon manually performed acetabular reaming and cup implantation with the target position on the navigation screen. There are variations in the manual procedures for nTHA, which could be why rTHA’s superiority over nTHA in cup positioning remains controversial.

Moreover, this study’s results showed a difference in the absolute error of postoperative LLD and offset discrepancy between the two groups. The absolute error of postoperative LLD was significantly smaller for rTHA than for nTHA, showing no consistent trend in the positive or negative direction of error, and was effective in reducing variability. The factors considered responsible for this are as follows: 1) the absolute cup positioning error in the SI direction of rTHA was small; 2) the robotic arm-assisted system shows the difference in leg length on the opposite side and the intraoperative change in leg length by measuring the position of the greater trochanter and the lateral femoral epicondyle with its navigation function; and 3) rTHA had a smaller absolute error in the acetabular offset reconstruction. Therefore, adjusting the head offset to optimize the global offset is unnecessary, which is beneficial for adjusting the LLD.

Kayani et al^31^ compared rTHA and manual THA and showed higher accuracy of cup position in rTHA. They reported no significant difference between rTHA and manual THA in postoperative LLD. On the other hand, in this study, the absolute value of postoperative LLD was significantly smaller in rTHA than in nTHA. In this study, 47 of 74 cases were DDH, with a mean preoperative LLD of 7.7 mm and a SD of 6.4 mm. Therefore, the required amount of leg lengthening consists of cup positioning, stem positioning, and head offset was large and variable in this study, which might have contributed to a more reduced postoperative LLD in rTHA than in nTHA.

The present study’s absolute errors of the postoperative acetabular, femoral, and global offsets were significantly smaller for rTHA than for nTHA, and there was no consistent trend in the positive or negative direction of the error. Two factors were considered to have reduced the variability in leg offset reconstruction in rTHA. First, the absolute cup positioning error in the ML direction of rTHA was small. The difference in the ML direction of the COR is directly associated with the acetabular offset. In all cases in this study, the cup was placed before the femoral stem. The global offset defines soft-tissue tension; hence, the surgeon ultimately determines the head offset, considering the soft-tissue balance.^4^ Therefore, the absolute error of the acetabular offset led to variations in the femoral offset, which affected the error in the global offset reconstruction. Second, the robotic arm-assisted system shows the difference in leg offset with the opposite side and the intraoperative change in leg offset with its navigation function, which helps surgeons determine the head offset. In this study, we assessed the clinical utility of global offset using a 5 mm threshold, as previous research indicated that an increase in acetabular offset of > 5 mm from the normal hip can lead to increased polyethylene wear.^32^ For rTHA, 95.8% of the cases exhibited a global offset discrepancy within 5 mm. However, for nTHA, only 68.2% achieved a global offset discrepancy of ≤ 5 mm, which was significantly lower than that observed in rTHA. Moreover, the number of both the postoperative LLDs and global offset discrepancies within approximately 5 mm was significantly higher in rTHA compared to nTHA. This result suggests that the accuracy of cup positioning in rTHA contributed to the reduced variability of postoperative LLD and global offset discrepancy compared with that in nTHA. However, further studies with a large number of participants, including gait analysis, physical activity analysis, and patient-reported outcomes, are warranted to assess the effect of reducing outliers within 5 mm in LLD and global offset discrepancy on clinical outcomes.

In comparing cup orientation, rTHA was significantly more accurate than nTHA for RI and RA, and the average RI and RA were larger for nTHA. Ando et al^16^ showed a similar result, with the absolute error in the RI relative to the target angle being smaller for rTHA and the average RA being larger for nTHA. Tamaki et al^18^ showed similar results for the absolute error of cup orientation for RI and RA. In nTHA, the final cup insertion was performed manually by checking the cup angle on the screen. Nishii et al^33^ reported that cup orientation fluctuates during press-fit cup fixation, even when using a navigation system. This study demonstrated that robotic arm control of cup orientation effectively prevents fluctuations in cup orientation while pushing the cup in the under-reamed acetabulum. The number of cases with cup orientation outliers greater than 5° from target was significantly lower in rTHA compared to nTHA. However, to assess the effect of reducing cup orientation outliers within 5° on the outcomes, a long-term follow-up study of a large number of cases is needed.

This study has some limitations. First, the sample size was relatively small; further analyses with more patients are necessary. Furthermore, examining the system in various institutions is crucial to evaluate the performance stability of a computer-assisted system regardless of the institution. In addition, this was not a randomized controlled trial. Propensity score matching was used to reduce the effects of age, sex, and BMI. However, case selection bias may still be present.

In conclusion, rTHA is more accurate in terms of cup orientation and position than nTHA, effectively reducing postoperative leg length and offset discrepancy.

**Take home message**

- Robotic arm-assisted total hip arthroplasty (rTHA) is more accurate in cup orientation and position than CT-based navigation THA.

- rTHA can effectively reduce postoperative leg length and offset discrepancy.

## Data Availability

The datasets generated and analyzed in the current study are not publicly available due to data protection regulations. Access to data is limited to the researchers who have obtained permission for data processing. Further inquiries can be made to the corresponding author.
